# Frequency-Dependent Modulation of Regional Synchrony in the Human Brain by Eyes Open and Eyes Closed Resting-States

**DOI:** 10.1371/journal.pone.0141507

**Published:** 2015-11-06

**Authors:** Xiaopeng Song, Shuqin Zhou, Yi Zhang, Yijun Liu, Huaiqiu Zhu, Jia-Hong Gao

**Affiliations:** 1 Department of Biomedical Engineering, College of Engineering, Peking University, Beijing, 100871, China; 2 School of Life Science and Technology, Xidian University, Xi’an, Shanxi 710071, China; 3 Center for MRI Research and Beijing City Key Lab for Medical Physics and Engineering, Peking University, Beijing, 100871, China; Southwest University, CHINA

## Abstract

The eyes-open (EO) and eyes-closed (EC) states have differential effects on BOLD-fMRI signal dynamics, affecting both the BOLD oscillation frequency of a single voxel and the regional homogeneity (ReHo) of several neighboring voxels. To explore how the two resting-states modulate the local synchrony through different frequency bands, we decomposed the time series of each voxel into several components that fell into distinct frequency bands. The ReHo in each of the bands was calculated and compared between the EO and EC conditions. The cross-voxel correlations between the mean frequency and the overall ReHo of each voxel’s original BOLD series in different brain areas were also calculated and compared between the two states. Compared with the EC state, ReHo decreased with EO in a wide frequency band of 0.01–0.25 Hz in the bilateral thalamus, sensorimotor network, and superior temporal gyrus, while ReHo increased significantly in the band of 0–0.01 Hz in the primary visual cortex, and in a higher frequency band of 0.02–0.1 Hz in the higher order visual areas. The cross-voxel correlations between the frequency and overall ReHo were negative in all the brain areas but varied from region to region. These correlations were stronger with EO in the visual network and the default mode network. Our results suggested that different frequency bands of ReHo showed different sensitivity to the modulation of EO-EC states. The better spatial consistency between the frequency and overall ReHo maps indicated that the brain might adopt a stricter frequency-dependent configuration with EO than with EC.

## Introduction

The state of having one's eyes open or closed has long been linked to changes in brain physiology. Different modalities like electroencephalography (EEG), magnetoencephalography, and functional magnetic resonance imaging (fMRI) have been applied to study the effects of opening or closing eyes on endogenous brain dynamics [1–3]. In blood-oxygen-level-dependent (BOLD) fMRI studies, both eyes-open (EO) and eyes-closed (EC) conditions have widely served as resting-states to characterize baseline activities or control state [4]. However, these two kinds of resting-states could have nontrivial differential effects on BOLD activities. The primary impact they have on brain dynamics is the alterations of brain rhythms. The EC condition is known to play a critical role in the generation of the alpha rhythm observed in EEG experiments [1]. Little is known about how BOLD oscillation frequencies would be modulated by these two states.

Beside the frequency of BOLD oscillations of a single brain region, the synchronization of BOLD activities of several regions that are anatomically distant or close can also be affected by the EO and EC states [5, 6]. Distant synchronization of BOLD activities is usually quantified by functional connectivity (FC), whereas local synchrony of the adjacent voxels can be characterized by regional homogeneity (ReHo) [7]. It is suggested that the regional synchrony might serve as the fundament of distant correlation: a brain area needs to build up local synchronization of neighboring neurons within itself before it interacts with other distant areas [8]. Therefore, before we study the frequency-specific changes of FC between EO and EC states, we attempted to clarify how regional synchrony through different rhythms was changed between the two states first in the current study.

Previous studies have found that, compared with the EC states, ReHo increased with EO in the bilateral occipital-parietal cortex, and decreased in the bilateral sensorimotor cortex, supplementary motor area (SMA), superior temporal gyrus (STG), and thalamus [5]. Although these studies revealed altered ReHo in multiple regions between EO and EC, it is unknown whether ReHo in different frequency bands was altered to the same extent or not. For instance, both the thalamus and the visual cortex may show significant changes of the overall ReHo between EO and EC, however whether these changes happen in the same frequency band or not is unclear. Previous studies suggested that different brain areas might recruit rhythms of distinct frequency bands to maintain local synchrony, and BOLD oscillations in different frequency bands may be related to assorted underlying cognitive processes [8–10], we hypothesized that ReHo in different frequency bands would behave differently in these areas between EO and EC.

In the resting-state human brain, the BOLD oscillation frequency of a single voxel and the regional synchrony of several neighboring voxels may not be independent. For instance, a certain neurophysiological process may affect a cluster of voxels and make them active in a synchronized way; also, this process may mainly contribute to the BOLD oscillations in a certain frequency band [9]. Hence these two measurements, though with different mathematical definitions and physiological implications, may partly share the same neurophysiological origins and interact with each other [9, 11]. Besides the shared dynamic neurophysiological processes, some structural factors such as the cytoarchitecture, synaptic low-pass filtering mechanism, and neurovascular coupling properties may also play important roles in constraining the relationship between BOLD frequency and regional synchrony [12]. However, few studies has quantified this relationship, and explored how this relationship varied across different brain areas and how it was modulated by different functional states of the brain. Clarifying this relationship may advance the understanding of how the brain is configured or confined by the spatial-varying and functional-related hemodynamic properties.

The purpose of the current study is hence to investigate the changes of BOLD dynamics (i.e. the oscillation frequency, ReHo, and their relationships) induced by opening or closing eyes. We intended to examine how the local synchronies through different frequency bands were altered by the two states.

## Material and Methods

Recently, several studies have successfully applied the Empirical Mode Decomposition (EMD) method to the analysis of the spectral characteristics of BOLD oscillations [9, 10]. The EMD automatically isolates the underlying processes of BOLD activities in a data-driven manner and divides the whole frequency band into adaptively determined sub bands without any assumption of linearity, stationarity, or recourse to any rigid priori chosen band-pass filter [9, 10].

There are several main reasons why we choose EMD to decompose the BOLD signal. First, the power spectrum of neuronal processes may spread over a certain frequency range with gradually decreasing power on both sides of the band-width. Band-pass filters, which impose sharp cutting-edges on the spectrum, could not preserve these spectral characteristics and may cause distortions [10], while EMD can isolate intact frequency components without changing their spectrum. Second, although previous literatures suggested that the slow resting-state brain rhythms could be further decomposed into slow waves of different frequency bands (slow-3, slow 4, and slow-5 etc.), we should note that these inferences were drawn from studies of mammalian forebrain (not human) and were derived from the fact that the mean frequency of the experimentally observed electrophysiological signals form a linear progression on a natural logarithmic scale [8]. It is still debatable whether these pre-defined bands should be applied to BOLD signals of the human brain. Moreover, applying the same rigid priori chosen band-pass filter to different brain areas or subjects fails to take into account of the spatial and cross-subject variations of the signal complexity. EMD can avoid these disadvantages, preserve individual features, and may get more biologically correct frequency band estimation. Third, in order to get more accurate estimation of the HWMF of the original BOLD signal we need to divide the whole band into narrower bands, and estimate the instantaneous frequency values of the narrowband signals, however band-passing a signal to achieve narrowband signals is counterproductive given the desired outcome of adaptively characterizing a signal and it would yield meaningless instantaneous frequency values [10]. Ideally, one needs to disentangle the different oscillatory components that make up the signal without distorting them, that is, preserving their nonlinear features. Only by using the EMD algorithm can we achieve this [10].

In the current study, EMD was applied in a voxel-wise fashion to adaptively decompose the BOLD time series of each voxel into several components called intrinsic mode functions (IMFs) [13]. Each IMF occupies a unique frequency range. We then calculated the following four metrics for each subject: (1) the Hilbert weighted mean frequency (HWMF) of the original BOLD signal of each voxel, (2) the overall ReHo of the original BOLD signal of each voxel, (3) the ReHo of each IMF of each voxel, (4) the cross-voxel correlation between the HWMF and the overall ReHo. For the consistency of terminology, the “overall ReHo” was used in the following context to address the ReHo of the original whole-band BOLD signal before EMD and to distinguish it from the ReHo in different frequency bands (IMFs).

### 2.1 MRI Data Acquisition

This work was approved by the Institutional Review Boards of Peking University and Beijing Normal University, and was in accordance with the Declaration of Helsinki. FMRI data were acquired from the open source website (http://fcon_1000.projects.nitrc.org/fcpClassic/FcpTable.html, Beijing: Eyes open Eyes Closed Study) provided by ‘1000 Functional Connectomes’ Project. Forty eight college students (aged 19–31 years, 24 female) were contained in the sample. No subject had a history of neurological, psychiatric, or medical conditions. Written, informed consent was obtained prior to scanning of all subjects.

Each participant underwent three resting state scanning sessions, each of which lasted for 8 min. Firstly an EC resting state session was scanned (data of this session was acquired for other purpose and not analyzed in the present study), followed by two sessions counter-balanced across subjects: one EO resting state and one EC resting state session. During all the sessions, participants were instructed to lie quietly in the scanner, not to fall asleep or think about anything in particular.

The MR images were acquired using a SIEMENS TRIO 3-Tesla scanner. The functional images were obtained by using an echo-planar imaging sequence with the following parameters: 33 axial slices, thickness/gap = 3.5/0.7 mm, in-plane resolution = 64 × 64, repetition time (TR) = 2000 ms, echo time (TE) = 30 ms, flip angle = 90°, field of view (FOV) = 200 × 200 mm^2^. Each condition consists of 240 functional volumes. In addition, a 3D T1-weighted magnetization-prepared rapid gradient echo (MPRAGE) image was acquired with the following parameters: 128 sagittal slices, slice thickness/gap = 1.33/0 mm, in-plane resolution = 256 × 192, TR = 2530 ms, TE = 3.39 ms, inversion time (TI) = 1100 ms, flip angle = 7°, FOV = 256 × 256 mm^2^.

### 2.2 Image Preprocessing

Images were analyzed by using the following procedure with SPM8 (http://www.fil.ion.ucl.ac.uk/spm). The first 10 time points were removed to eliminate non-equilibrium effects of magnetization. The remaining 230 volumes of functional BOLD images were corrected for slice timing effects, motion corrected and spatially normalized to the Montreal Neurological Institute (MNI) template using the standard EPI template, resulted in functional image series of 61×73×61 voxels (voxel size of 3 mm × 3 mm × 3 mm). No translation or rotation parameters in any given data set exceeded ±2 mm or ± 2 degree. These images were not spatially smoothed as previous studies suggested [7]. Motion parameters, linear trend, and signals from the ventricles and white matter, were regressed out from each voxel’s time course, to correct for co-fluctuations in BOLD signal due to noise.

### 2.3 Empirical mode decomposition and Hilbert weighted mean frequency

The EMD method [13] decomposes the original BOLD signal of each voxel into a finite set of intrinsic oscillatory components, termed IMFs. Each IMF occupies a unique frequency range: the first IMF occupies the highest frequencies, and the last occupies the lowest, with the other IMFs in between. Mathematically, for a real-valued BOLD signal *x*(*t*), the standard EMD finds a set of *K* IMFs {*IMF*_*i*_(*t*)}, *i* = 1 to *K*, and a monotonic residue signal *r*(*t*), so that

$$x\left(t\right)=\overset{K}{\underset{i=1}{\sum }}IMF_{i}\left(t\right)+r\left(t\right)$$ (Eq 1)

To ensure that the time frequency spectra yields meaningful frequency estimates (e.g. no negative frequencies), IMFs{*IMF*_*i*_(*t*)} are defined so as to have symmetric upper and lower envelopes, with the number of zero crossings and the number of extrema differing at most by one. To extract IMFs using EMD, an iterative method known as sifting algorithm is used; for illustration, a sifting procedure for obtaining the first IMF (IMF1) from the signal *x*(*t*) is outlined in the algorithm below.

The standard EMD algorithm:

1. Find the locations of all the extrema of *x*(*t*);
2. Interpolate between all the minima (resp. maxima) to obtain the lower (resp. upper) signal envelope, *elow*(*t*) (resp. *eup*(*t*));
3. Compute the local mean time course *emean*(*t*) = [*elow*(*t*)+*eup*(*t*)]/2;
4. Obtain the “oscillatory mode” from *r*(*t*) = *x*(*t*)−*emean*(*t*);
5. If *r*(*t*) obeys the stopping criteria, *IMF*_*i*_(*t*) = *r*(*t*) becomes an IMF, otherwise set *x*(*t*) = *r*(*t*) and repeat the process from Step 1.

To obtain remaining IMFs, the same procedure is applied iteratively to the residual *r*(*t*) = *x*(*t*)−*IMF*_*i*_(*t*) until we are left with the monotonic signal. The standard stopping criterion terminates the sifting process only after the IMF condition is met for *S* consecutive times (*S* is normally taken to be 2 or 3), here S = 3.

The Hilbert weighted frequency (HWF) [14] of each IMF was determined using instantaneous information about amplitude and phase to reflect the mean oscillation frequency of the IMF. The HWFs of all the IMFs of one voxel were then weighted by the norm value of each IMF, summed, and then divided by the sum of the norm values of all the IMFs to get the HWMF for the voxel. HWMF represents the overall frequency of the original time course of the voxel. The HWMF considers the contributions of all the IMFs and the IMFs with higher energy (or norm) contribute more to the mean frequency. Biologically the HWMF represents how fast the original BOLD oscillation is. Both the HWF and the HWMF are comparable to the traditional Fourier frequency and are measured by units of Hertz. See Supporting Information **S1 Appendix** for the discussion of how to calculate HWF and HWMF [14]. For almost all the voxels in the brain, the decomposition of the time course yielded five IMFs, denoted as IMF1 to IMF5, and the frequency range of the first five IMFs covers 0–0.25 Hz. In very rare cases, voxels near the surface of the brain or inside the ventricle yielded more than five IMFs due to inevitable noise. In these cases only the first five IMFs were considered.

### 2.4 Regional homogeneity

After finishing the aforementioned preprocessing steps, the original time course of each voxel was decomposed into several IMF time courses. The original time course and the first five IMFs were ready for ReHo analyses. Data were analyzed using the resting-state fMRI Data Analysis Toolkit (http://www.restfmri.net/forum/index.php). ReHo analysis was performed for the original time course (0–0.25 Hz, without band-pass filtering) and each IMF of each intracranial voxel by calculating the Kendall’s coefficient of concordance (KCC) of the respective time series of the voxel with those of its nearest neighbors (26 voxels) in a voxel-wise fashion:

$$W=\frac{\sum {\left(R_{i}\right)}^{2}-n{\left(\bar{R}\right)}^{2}}{1/12k^{2}\left(n^{3}-n\right)}$$

Where *W* ranges from 0 to 1; $R_{i}=\sum_{j=1}^{k}r_{ij}$ where *r*_*ij*_ is the rank of the *i* ^th^ time point in the *j* ^th^ voxel; $\bar{R}=\left(n+1\right)k/2$ is the mean of the *R*_*i*_, *n* is the number of time points of each IMF time series (here n = 230), and k is the number of time series within the measured cube of voxels (here k = 27, the central voxel plus its 26 neighbors). Thus, we obtained the overall ReHo value for the original time course and one ReHo value for each of the first five IMFs of the voxel.

### 2.5 Statistical analysis

The individual Frequency maps and ReHo maps (including the overall ReHo calculated with the original time course and the ReHo calculated with each of the IMFs) were smoothed with a Gaussian kernel of 4 mm full width at half-maximum. All the frequency maps and ReHo maps were transformed to z-score maps. Paired *t*-tests were performed on the frequency and ReHo z-score maps in a voxel-wise manner to reveal the differences of frequency, overall ReHo, and the ReHo in different frequency bands between EO and EC. AlphaSim corrections for multiple comparisons were executed with a voxel-level threshold of *p* < 0.01 and a minimum cluster size of 68 voxels, yielding an overall false positive *P* < 0.01.

Areas with significant changes of frequency or overall ReHo were selected as ROIs. To investigate in what frequency bands the ReHo showed the most significant changes, the mean ReHo of different IMFs (frequency bands) in these ROIs were calculated by averaging the ReHo values of all the voxels within these ROIs respectively. Paired *t*-tests were carried out to compare the mean ReHo in different frequency bands in these ROIs between EO and EC.

We hypothesized that voxels with lower BOLD oscillation frequency would show higher regional synchronization with its neighboring voxels, also the relationship between the frequency and the overall ReHo varied across the brain. To test this hypothesis, subject brains registered to standard MNI space were divided into 116 regions using the standard Automated Anatomical Labeling (AAL) atlas. Cross-voxel correlation analyses between the frequency and overall ReHo were performed within each of the 116 regions. To show that the frequency-overall ReHo correlations in some brain areas were also modulated significantly by the EO-EC conditions, the cross-voxel correlation coefficients within these AAL regions under these two conditions were transferred to z-scores and compared by using paired *t*-tests.

## Results

### 3.1 Eyes-open (EO) and eyes-closed (EC) states modulate ReHo in specific frequency bands

Each IMF occupies a unique frequency band with very slight overlap: the HWF of the first IMF (IMF1) occupies the highest frequency band (0.1–0.25 Hz); the HWF of IMF2 of all the voxels range from 0.04 to 0.1 Hz; IMF3 from 0.02 to 0.04 Hz; IMF4 from 0.01 to 0.02 Hz; and IMF5 occupies the lowest frequency band 0 to 0.015 Hz. The HWMF values of the original BOLD time courses range from 0.03 to 0.11 Hz (See Supporting Information **S1 Fig** for the distribution histograms of HWF values of each IMF and the histogram of HWMF values of the original BOLD time courses of all the voxels in the brain). Supporting Information **S2** and **S3 Figs** show the group-mean HWMF maps across the 48 subjects with EO and EC respectively. Results showed that, with either EO or EC, the HWMF was lower in the posterior cingulate, precuneus, bilateral and medial prefrontal cortices, and bilateral inferior parietal lobules. HWMF was higher in the sensorimotor network, bilateral temporal cortices, and subcortical regions. The frequency range covered by each IMF was consistent across EO and EC states (See Supporting Information **S4 Fig** for the group averaged power spectrum of each IMF), and there was no significant difference of HWF of each IMF between EO and EC.

**Fig 1:**
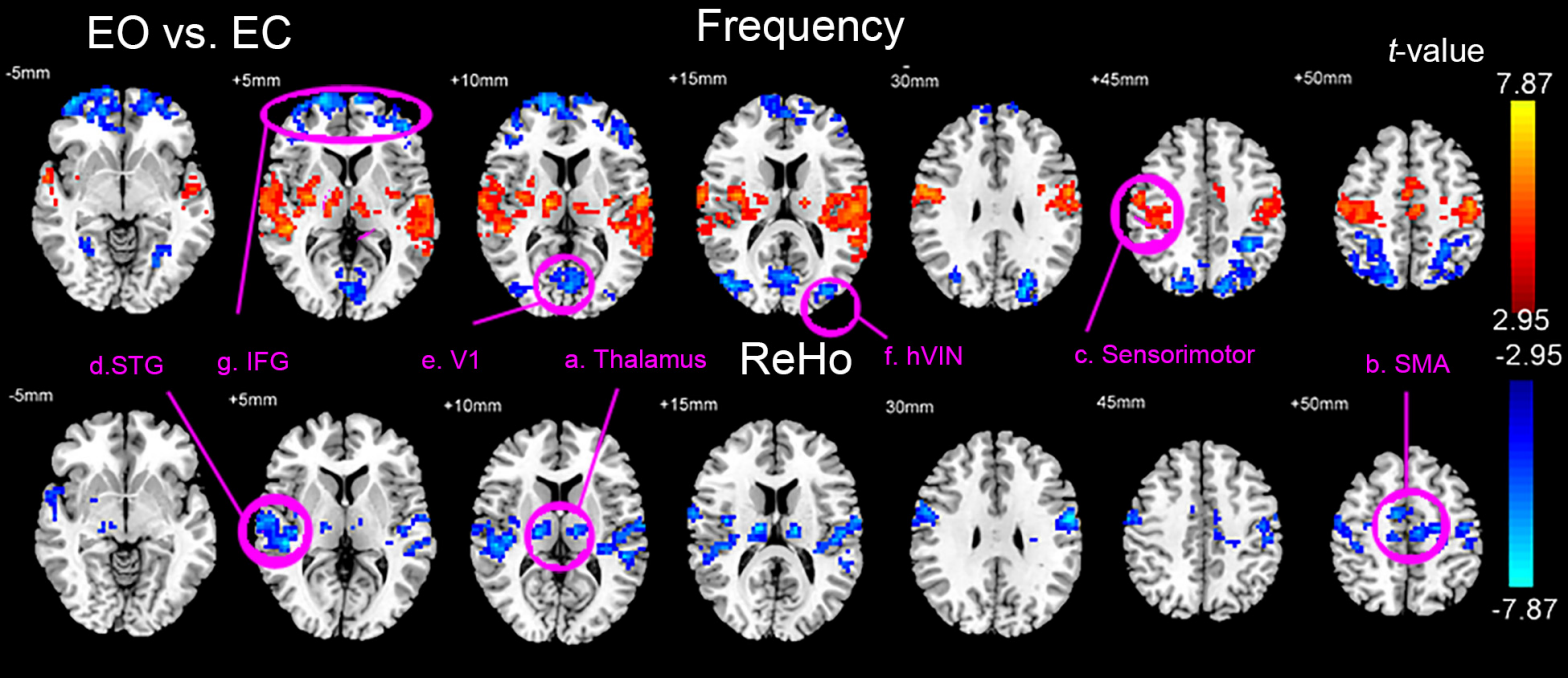
Brain areas with significant changes of frequency and overall ReHo. Results of the paired *t*-tests (P<0.01, Alphasim corrected) of Frequency (upper panel) and the overall ReHo (lower panel) between EO and EC conditions. The areas selected as ROIs are marked and labeled: a. Thalamus, b. Supplementary motor area (SMA), c. Sensorimotor cortex, d. Superior temporal gyrus (STG), e. Primary visual area (V1), f. High-order visual network (hVIN), g. Inferior frontal gyrus (IFG).

**Fig 1** and Supporting Information **S1 Table** show the results of paired *t*-tests (EO vs. EC, AlphaSim corrected, voxel-level *p* < 0.01, cluster size > 68 voxels, overall false positive *P* < 0.01) of the frequency and the overall ReHo between EO and EC conditions. The results showed that, compared with the EC state, during the EO state, the bilateral thalamus (mainly the ventral lateral nucleus), supplementary motor area (SMA), sensorimotor cortex, and superior temporal gyrus (STG) showed significantly higher frequency but lower overall ReHo. The primary visual cortex (V1), higher order visual network (hVIN, consists of bilateral middle occipital gyrus and superior parietal gyrus), and bilateral inferior frontal gyrus (IFG, also includes part of the medial prefrontal cortex and orbital frontal cortex) showed significantly lower frequency with no significant changes of overall ReHo.

**Fig 2:**
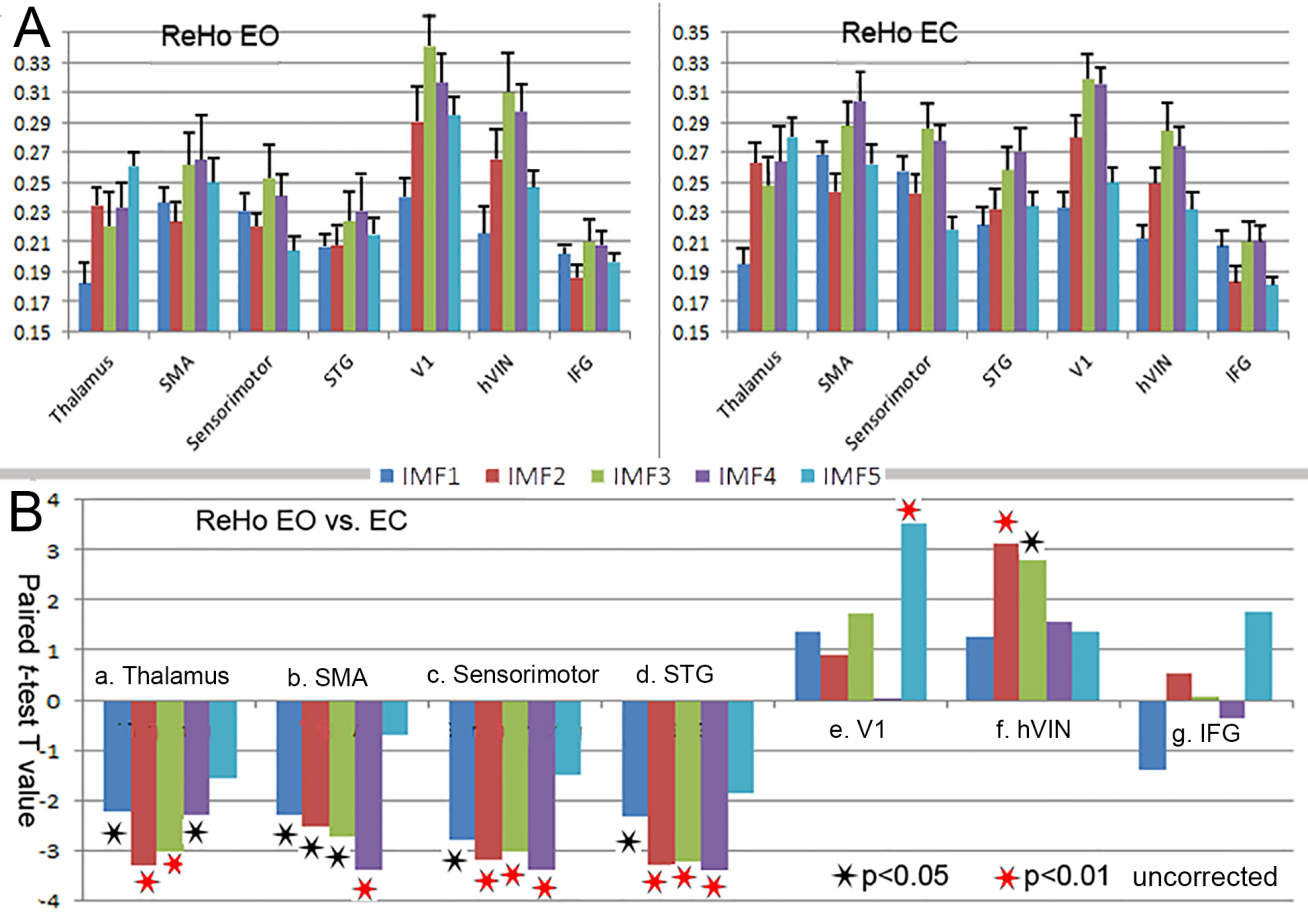
Frequency specific changes of ReHo in the ROIs. **(A)** The group-average distribution of ReHo across different frequency bands (IMFs) with EO (left panel) and EC (right panel). (**B)** Results of paired *t*-tests (EO vs. EC) of ReHo in the five frequency bands (IMFs) in the eight ROIs. Black and red asterisks indicate significance levels of p<0.05 and P<0.01 respectively.

The above seven brain areas, four with significant changes in both the frequency and the overall ReHo and three with significant changes in the frequency only, were selected as ROIs. **Fig 2A** shows the group-average distribution of ReHo across different frequency bands (IMFs) within these ROIs. Results showed that the distribution patterns of ReHo were distinct for different brain areas. In THA the ReHo values in IMF5 (0–0.01 Hz) were the highest among the five IMFs, followed by IMF2 (0.04–0.1 Hz) and IMF4 (0.01–0.02 Hz). In SMA and sensorimotor cortex, ReHo values in IMF3 and IMF4 were the highest followed by IMF1 (0.08–0.25 Hz). In V1 and hVIN, the ReHo values in IMF3 (0.02–0.04 Hz) were the highest among the five IMFs, followed by IMF4 (0.01–0.02 Hz). STG and IFG also showed distinct frequency-dependent ReHo signatures. These signatures were consistent across both EO and EC sessions.

**Fig 2B** shows the results of paired *t*-tests of ReHo in the five frequency bands (IMFs) for each ROI. Compared with the EC state, during EO state, ReHo mainly decreased in IMF2 and IMF3 in the thalamus (*p* < 0.01); for the SMA, ReHo mainly decreased in IMF4 (*p* < 0.01); for the sensorimotor cortex and STG, ReHo mainly decreased through IMF2 to IMF4 (*p* < 0.01). In the V1, ReHo increased significantly in IMF5 (*p* < 0.01). For the hVIN, ReHo increased significantly in IMF2 and IMF3 (*p* < 0.01). For the IFG, ReHo showed no significant changes in any of the five IMFs.

**Fig 3:**
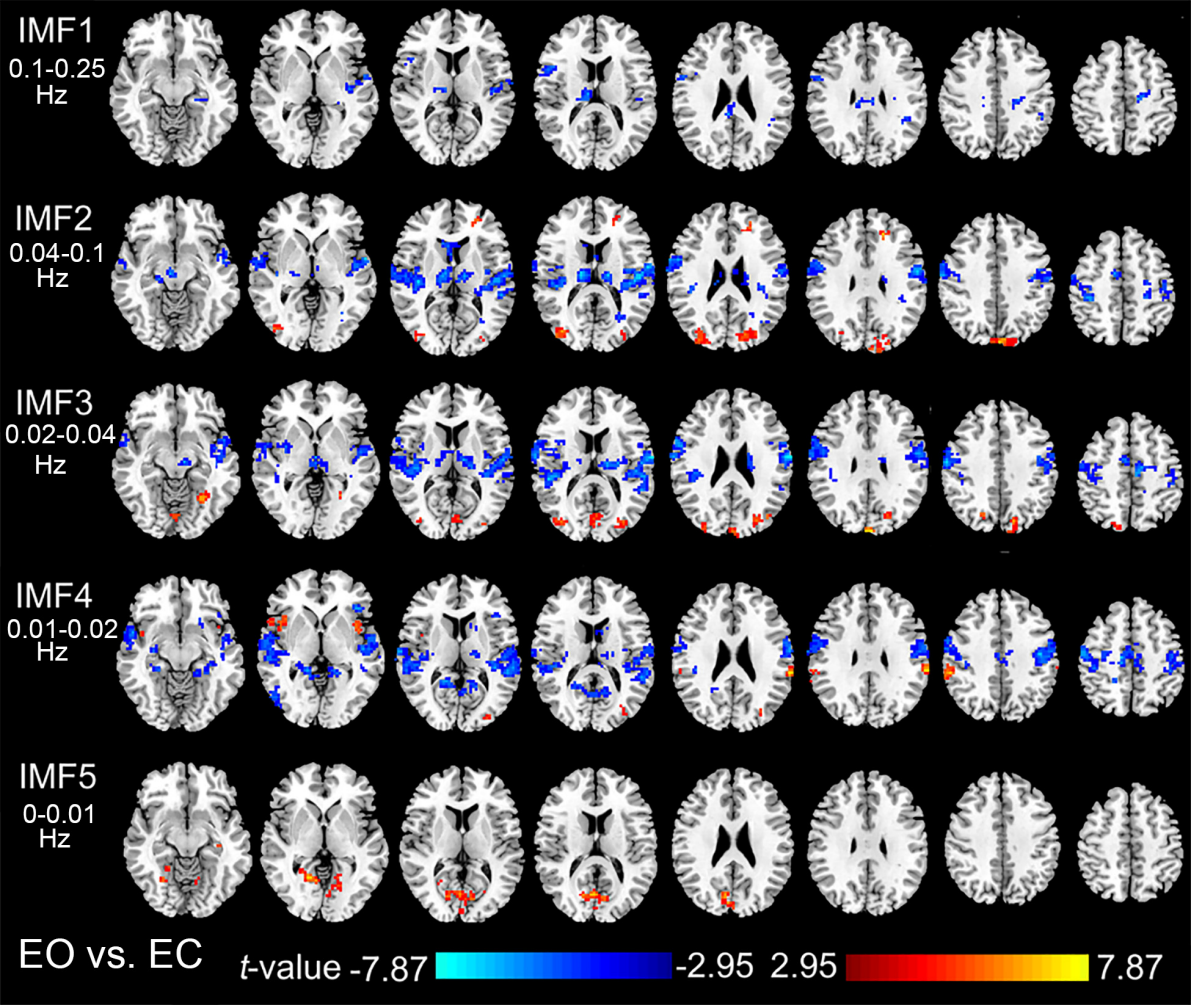
Paired t-tests of ReHo in different frequency bands. Results of the paired *t*-tests (P<0.01, Alphasim corrected) of the ReHo maps in the five IMFs between EO and EC conditions.

**Fig 3** shows the results of paired *t*-tests (EO vs. EC, AlphaSim corrected, voxel-level *p* < 0.01, cluster size > 68 voxels, overall false positive *P* < 0.01) of the ReHo in the five frequency bands (IMFs). Consistent with the results of the ROI analysis, the voxel-wise paired *t*-tests showed significant decreased ReHo in the first four IMFs in the THA, SMA, sensorimotor cortex, and STG. The V1 showed significant increased ReHo in IMF5, while the hVIN showed significant increased ReHo in IMF2 and IMF3.

### 3.2 EO-EC states modulate the cross-voxel correlation between Frequency and overall ReHo

**Fig 4:**
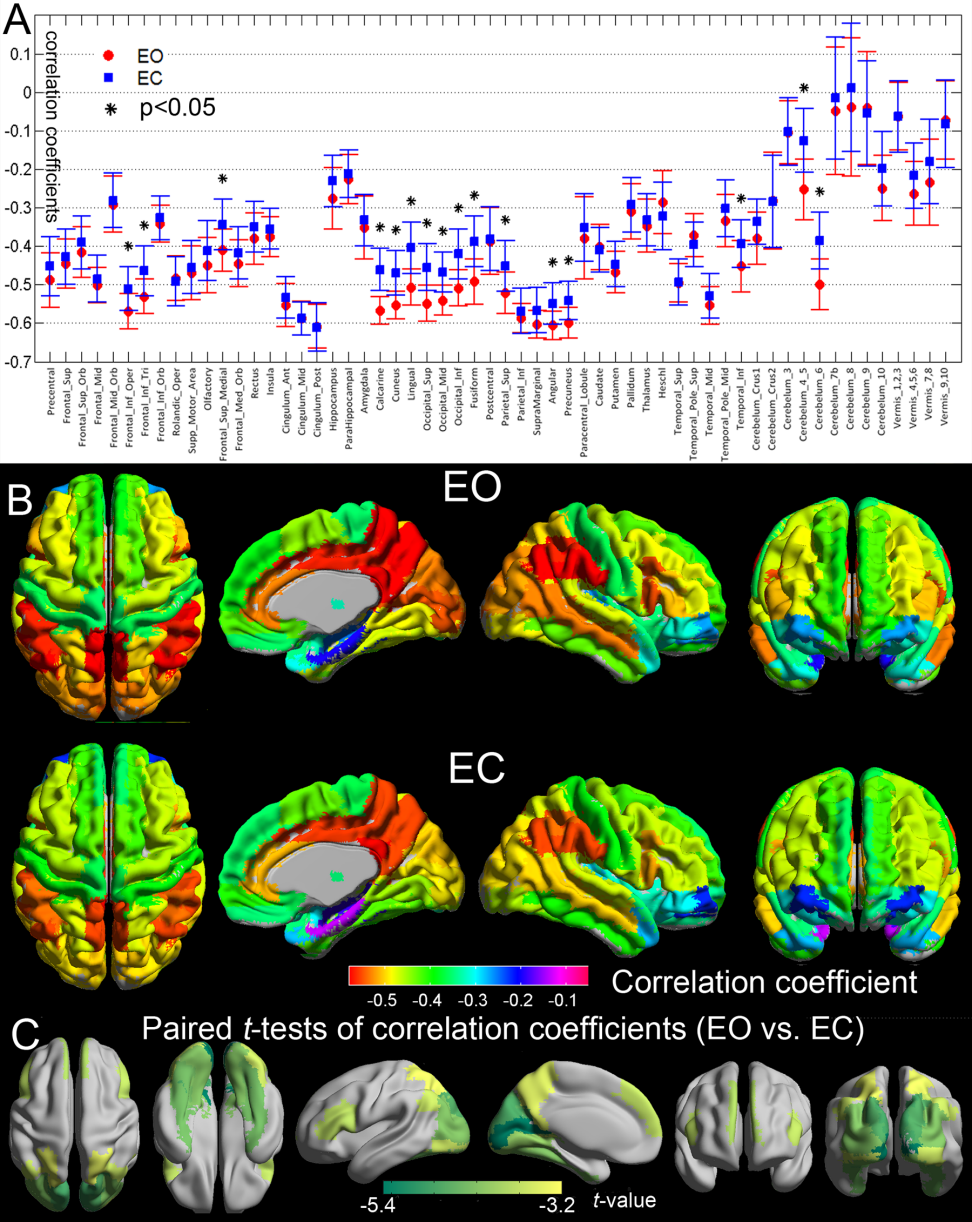
Frequency-ReHo correlations in different brain regions. (**A)** Mean and standard deviations (SD) values of cross-voxel correlation coefficients within each AAL region under the condition of EO and EC. Cross-voxel correlation coefficients between Frequency and ReHo were calculated within each AAL region of EO and EC respectively for each subject. Group mean and SD of correlation coefficients for were then determined across the 48 subjects. Brain areas with significant changes of correlations coefficients were marked with asterisks. (**B)** Group-mean maps of frequency-ReHo correlation coefficients of different AAL regions under EO and EC. The same scaled color bar is used for both EO and EC conditions. (**C)** Maps of AAL regions with significantly changed frequency-ReHo correlation. Regions with significant changes of frequency-ReHo correlations were labeled with different colors representing the *t*-values.

**Fig 4A** shows the cross-voxel correlation between Frequency and overall ReHo within different AAL regions under the condition of EO and EC, brain areas with significant changes of frequency-overall ReHo correlations between EO and EC were marked with an asterisk. **Fig 4B** renders the same results in a more intuitive way. AAL regions were labeled with different colors representing the frequency-overall ReHo correlation coefficients. In both of the EO and EC states, the frequency-overall ReHo coupling in the bilateral inferior frontal gyrus, the cingulate gyrus, the inferior parietal lobule, the supramarginal gyrus, the angular gyrus, and the precuneus stronger (higher absolute values of correlation coefficients) compared with that in the other brain areas. **Fig 4C** shows the results of the paired *t*-tests of the frequency-overall ReHo correlations for different AAL regions in a more intuitive way, brain areas with significant changes of frequency-overall ReHo correlations were labeled with different colors representing the t-values. Compared with the EC state, the frequency-overall ReHo correlations were stronger with EO in the occipital lobe, the inferior frontal gyrus, medial prefrontal cortex, superior parietal lobule, angular gyrus, precuneus, inferior temporal gyrus, and parts of the cerebellum.

## Discussion

Multiple ongoing neural processes may co-exist in the same area within a small volume and be maintained by brain waves of different frequencies [8], these processes may be reflected by BOLD oscillations in different frequency bands [9, 11]. These processes of different frequency bands may be related to assorted underlying cognitive functions, and might be the biological basis for the multiple functions of a certain functional brain area [8]. These rhythms can further be modulated by or show different sensitivity to different tasks, stimuli or pathophysiological states. The EMD is a useful method to automatically isolate these underlying processes [9]. Hence the biological implications of the IMFs might be the underlying neural processes of different functions. Compared with the IMFs, the raw BOLD time course can be seen as a mixture of these different processes. By interrogating the sensitivity of different IMFs to the changes of EO and EC states, the current study attempted to test these hypotheses. Still further studies and experiments are needed to help us understand the exact biological meanings of different brain rhythms.

In the present study, we investigated the relationships between BOLD oscillation rhythms and ReHo in two aspects: the distribution of ReHo across different frequency bands; and the correlation between frequency and the overall ReHo. We showed that both aspects were affected by structural and functional variables. The distribution profiles of ReHo across the spectrum and the correlation coefficients between frequency and the overall ReHo varied from region to region, reflecting the anatomical or structural significance of the relationships between brain rhythms and regional synchrony. Also these relationships were modulated significantly by the EO-EC states, reflecting the functional significance of these relationships.

### 4.1 Frequency specific changes of ReHo

Consistent with previous findings [5, 6], we have found significantly increased frequency and decreased ReHo in the bilateral thalamus, SMA, sensorimotor cortex, STG, and insula during the EO state. These areas are related to somatosensory, auditory, and vestibular functions, as well as interoceptive awareness. The bilateral primary visual cortex, middle occipital gyrus, superior parietal gyrus, and IFG showed significantly decreased frequency and increased ReHo in specific frequency bands. These areas are related to visual processing and attention. These results suggest that the EO state is an “exteroceptive” state characterized by attention and ocular motor activity, while the EC state is an “interoceptive” state characterized by internal integration and multisensory activity.

Although previous studies have reported similar results by using ReHo [5, 15], this is the first study to show frequency-specific changes of ReHo between EO and EC states. Our previous study has shown that different brain areas have distinct frequency-dependent ReHo signatures [9]. Although the ReHo in specific frequency bands were modulated significantly by EO and EC states, the relative profiles of the distribution patterns of ReHo of a certain region remained similar under these two conditions. The distinct profiles of ReHo distribution over the frequency bands assigned each brain area a unique frequency-specific ReHo signature which is stable and consistent across both EO and EC sessions. The frequency-specific ReHo signature might be constrained by the cytoarchitecture, synaptic types, and neurovascular properties of the local brain tissue. Since these properties were the structural properties of neural assemblies, they might not be affected significantly by different functional states [9].

The bilateral thalamus, SMA, sensorimotor cortex, and STG showed significantly higher overall ReHo in EC than in EO (**Fig 1B**), however different frequency bands contributed to the changes of ReHo quite differently (**Fig 2B** and **Fig 3**). In the thalamus, ReHo of the IMF2 showed the most significant changes, while in the cortical areas, ReHo of relatively slower frequency band, the IMF4, showed the most significant changes. Various fMRI, MEG, and electrophysiological studies have suggested that more complex neural structures and higher order functions tended to be associated with faster brain rhythms [10, 12, 16–18]. This general principle also applies to our findings. The thalamus is a complex structure with manifold functions of relaying of sensory and motor signals to the cerebral cortex, and the regulation of consciousness, sleep, and alertness. The ReHo of faster rhythms in this complex structure might be more sensitive to the changes between EO and EC states, while for the cortical areas, the ReHo of slower rhythms might be more sensitive.

The overall ReHo failed to detect the physiological changes in the V1, hVIN, and IFG between EO and EC states. In spite of this, we found that the BOLD oscillation frequency in these areas were significantly lower with EO, moreover the V1 showed significantly increased ReHo in IMF5, and the hVIN showed significantly increased ReHo in IMF2 and IMF3. In fact, one previous study has reported increased overall ReHo in the visual areas with EO [5], however in that study a lower threshold for significance level was used. These results suggested that the BOLD oscillation frequency can be more sensitive to neurophysiological changes in different brain states than the overall ReHo. The changes of ReHo in a few frequency bands were not effective enough to make the overall ReHo change significantly. Analyzing the changes of ReHo in detailed frequency bands would result in higher sensitivity and specificity, and provide more information than simply analyzing the overall ReHo.

It is not likely that the increased ReHo and the decreased frequency in the visual areas were induced by passive visual input, since previous studies have shown that the marked effects on the dynamics and connectivity of fMRI time series brought by volitional eyes open or closed are simply endogenous and irrespective of exogenous visual inputs [19]. The increased ReHo of IMF5 in the V1 might reflect endogenous changes of neurophysiological processes brought by EO, when extra visual stimuli were imposed, ReHo in the other frequency bands may be modulated [20]. This increased low-frequency ReHo in V1 may also reflect increased regional blood perfusion. A recent study reported that the only region showing significant changes of mean CBF between EO and EC states is the V1 area [2], we speculated that CBF may be more closely associated with low-frequency BOLD oscillations than with high frequency oscillations. Further fMRI studies are needed to confirm this supposition. Since IFG has been typically implicated in executive control, EO may elicit a status of higher alertness with readiness and expectation to see [21, 22], which could be associated with lower oscillation frequency in the IFG, and visual and ocular motor systems.

### 4.2 Regional specific changes of the Frequency-overall ReHo relationships

The distribution of the HWMF has a clear boundary between 0.03 and 0.11 Hz (Supporting Information **S2 Fig**), which is quite similar to the conventional 0.01–0.1 Hz frequency band of interest in resting-state community [9]. Voxels with lower HWMF also showed higher ReHo. These results indicated that low-frequency BOLD fluctuations contained more energy and were more likely to reflect active neuronal processes than the high-frequency BOLD fluctuations. Although the conventional preprocessing steps, such as detrending or band-pass filtering the raw BOLD signal, may lose some details, most of the information about neuronal synchrony could be preserved in the band of 0.01–0.1 Hz.

The negative correlations between BOLD oscillation frequency and regional homogeneity demonstrated a general rule of brain organization. Voxels with slower BOLD oscillations exhibited higher temporal synchronization with their neighboring voxels. The synaptic low-pass filtering mechanism may account for this phenomenon. Baria et al. has proposed a mathematical model to explain the correlation between functional connectivities and BOLD frequencies [12]. According to this model, the inputs into a neural ensemble could be broadband, however if we assume that the activity of a neural ensemble represents effectively a low-pass filter of its inputs, the relative spectral content of its output will be highly colored towards lower frequencies as a function of its connectivities with the adjacent neural ensembles. As a result, brain regions with more synaptic connections tend to have higher local synchronization and lower frequency [12].

The structural and functional significance of the frequency-overall ReHo interrelationship can be indicated by regional variation and the EO-EC states modulation. The correlation between frequency and the overall ReHo varied from region to region, reflecting the anatomical or structural significance of this relationship. Besides the synaptic low-pass filtering mechanism, other factors may influence the spectrum of neural dynamics, such as neurovascular coupling properties, brain metabolic processes, and even some non-neuronal physiological changes [23, 24]. These factors may vary from region to region, affect both the frequency and local synchronization of BOLD activities, and confined the frequency-overall ReHo relationship. **Fig 2C** shows that for all the cortical areas, IMF3 and IMF4 have the highest ReHo. We speculated that the underling neural processes could induce higher regional synchrony in BOLD signal, and because of the signal transduction mechanisms from electrophysiology to hemodynamics (or some other mechanisms), these processes were mainly reflected by BOLD oscillations in IMF3 and IMF4 bands. For different brain areas, those areas with higher neural activities would show higher energy in IMF3 and IMF4, which led to lower HWMF, meanwhile these areas would exhibit higher overall ReHo than the other areas, hence led to a cross-area correlation between HWMF and the overall ReHo.

Regional CBF has been shown to be highly correlated to functional connectivity strength across the whole brain, indicating local metabolism is higher in brain regions that have overall stronger functional connections [25, 26]. Moreover, some studies demonstrated that this metabolism-connectivity correlation was greater in brain regions associated with higher-order information processing [25, 26], which directly parallels our results showing stronger frequency-overall ReHo correlations in the bilateral IFG, the cingulate gyrus, the inferior parietal lobule, and the precuneus. Based on these findings, it makes sense to suggest that BOLD oscillation frequency might also be a marker of regional metabolic energy consumption [25, 26]. In the current study, the better spatial consistency between the frequency and the overall ReHo maps under the EO condition implied that the brain might adopt a stricter or more controlled frequency-dependent configuration that the brain tended to allocate higher regional synchrony or more energy to areas with slower rhythms with EO.

We speculated that the frequency-overall ReHo relationship could be an indicator of brain states. We found that BOLD oscillation frequency more closely reflects the overall ReHo during EO state than during EC state, indicating that the hemodynamic activities under the EO state are more constrained or predictable than under the EC state. Previous studies have found that compared with the EC states, the EO states were associated with decreased BOLD signal variance, fractional amplitude of low frequency fluctuation, and Hurst exponent in widespread cortical and subcortical regions [19]. Further, the hemodynamic activities of task-state might be even more constrained than that of the EO resting-state [12][27], and during drowsiness or slow wave sleep the brain would be less constrained than the EC resting-state [28] [29]. The more stringent relationship between BOLD oscillation frequency and overall ReHo during EO may indicate a more constrained or controlled state. At this stage these are merely speculations, examining the frequency-overall ReHo relationship with task-based fMRI experiments and neuroimaging data of different levels of consciousness would provide a clearer picture of the implications of this relationship and its dependence on brain states.

The EO-EC states modulated the frequency-overall ReHo relationship significantly in the inferior frontal gyrus, medial prefrontal cortex, superior parietal lobule, precuneus, and inferior temporal gyrus. These areas are thought to be the key nodes of the default mode network (DMN) which plays important roles in “mind wandering” [30], sleep [31], and the modulation of EO-EC states as well [32]. A previous study found that occipital alpha power was positively correlated with DMN BOLD activity only with EO but not with EC, implying that the coupling between EEG alpha rhythms and DMN BOLD oscillations might serve to block external visual input from interfering with introspective mental processing with EO, while under the EC condition, the lack of external visual input renders such a gating mechanism unnecessary [32]. Although in the current study the frequency and ReHo were not significantly changed in the DMN, the alterations of the frequency-overall ReHo relationships in DMN may still indicate some kind of implicit changes of the frequency-dependent resource allocation and the modulation of internal cognitive states.

In the current study, the two measurements, frequency and the overall ReHo, agreed better with each other in EO. In this sense, studies using EO state as the controlled resting-state may get more consistent results from different measurements. Several previous studies suggested that having subjects keep their eyes open (with or without fixation) rather than closed resulted in the higher reliability and consistency of FC both within and across subjects in the resting-state networks [4]. Recent studies using both BOLD and cerebral blood flow (CBF) imaging contrasts also observed that reliabilities of the EO state were higher than those of the EC state [2, 33]. These findings indicate that eyes-open resting may be a more favorable condition to conduct brain resting state fMRI and to serve as a controlled state [24]. The higher reliability of the EO state may partly result from the suppression of respiration-related influences [23, 24]. Previous studies have observed significant correlations among the fluctuation of respiration, the changes of global alpha EEG power, and BOLD oscillations in cortical areas at resting state, suggesting a mutual link of neuronal origin between the alpha EEG power, respiration, and BOLD dynamics. In particular, this correlation is much stronger in EC than in EO [24]. Another study also found significantly higher correlation and shorter latency between the partial pressure of end-tidal CO_2_ and BOLD signals in the EC condition compared with the EO condition [23].

## Conclusion

The current study has explored the physical and physiological properties of local resting-state hemodynamic activities from a new point of view. We demonstrated that human brain adopts distinct frequency-dependent configurations under EO and EC states and the different measurements agreed with each other better with EO, suggesting that the EO state might be a more favorable condition to serve as a controlled baseline state. To the best of our knowledge, this is the first study to clarify the frequency specific changes of ReHo, and to quantify the relationship between frequency and ReHo under different resting-states. Our study may provide useful information for further resting-state studies and fMRI experimental design.

## Supporting Information

S1 Appendix Hilbert Transform and the calculation of HWF and HWMF. (DOCX)

S1 Fig The histograms of HWF values of each IMF and the histogram of HWMF values of the original BOLD time courses of all the voxels in the brain. The histograms of HWF of IMF1 to IMF5 (color-coded by red, blue, green, pink, and cyan respectively) were determined from all the voxels in the whole brains across all the 48 subjects and across both the EO and EC conditions. Heights of the histograms represent the percentage of voxels in the whole brain whose HWF equals that frequency on the horizontal axis.
(TIF)

S2 Fig The group-mean HWMF map across the 48 subjects with eyes open. Voxels whose HWMF are equal or less than 0.04 Hz are color-coded as dark blue, voxels whose HWMF are equal or higher than 0.80 Hz are color-coded as dark red.
(TIF)

S3 Fig The group-mean HWMF map across the 48 subjects with eyes closed. Voxels whose HWMF are equal or less than 0.04 Hz are color-coded as dark blue, voxels whose HWMF are equal or higher than 0.80 Hz are color-coded as dark red.
(TIF)

S4 Fig The average power spectrum of each IMF for the seven ROIs. The IMF time courses of all the voxels within each of the ROIs were extracted and averaged to get a mean IMF time course from which the power spectrum of that ROI of that subject was calculated. For each IMF of each ROI, the power spectrums were then averaged across the 48 subjects.
(TIF)

S1 Table Brain areas with significant changes of Frequency or ReHo between EO and EC conditions. Alphasim corrected, voxel-level p < 0.01, cluster size > 68 voxels, overall false positive P < 0.01. Brain areas with significant changes of HWMF and ReHo are labeled with orange and green backgrounds respectively.
(DOCX)

## Abbreviations

ReHo: regional homogeneity
EMD: Empirical Mode Decomposition
HWF: Hilbert weighted frequency
HWMF: Hilbert weighted mean frequency
IMF: intrinsic mode function
SMA: supplementary motor area
STG: superior temporal gyrus
V1: primary visual cortex
hVIN: higher order visual network
IFG: inferior frontal gyrus
