# Supplementary material for: Frequency-Dependent Modulation of Regional Synchrony in the Human Brain by Eyes Open and Eyes Closed Resting-States
Source: PLoS One. 2015 Nov 6;10(11):e0141507. doi: 10.1371/journal.pone.0141507 (PMC4636261; doi:10.1371/journal.pone.0141507)
Supplement: S1 Appendix — (DOCX) [file pone.0141507.s001.docx]

### Hilbert transform

After the extraction of IMFs from original signal, say$X\left( t \right)$ is one of them, using HT, we can perform its Hilbert Transform $Y\left( t \right)$ using:

$Y\left( t \right)=\frac{1}{\pi}P\int\frac{X\left( t‘ \right)}{t-t’}dt$ (Eq.1)

Where 𝑃 is the Cauchy principle value:

$P\int_{\alpha}^{\beta} f(x)dx=\lim_{\varepsilon\to\infty} (\int_{\alpha}^{\xi-\varepsilon} f(x)dx+\int_{\xi+\varepsilon}^{\beta} f(x)dx)$ (Eq.2)

Using this definition, both *X*(𝑡) and 𝑌 (𝑡) can be combined to form an analytic signal, 𝑍(𝑡), given by:

$Z(t) = X(t) + iY(t) = {a(t)}^{ei\theta(t)}$ (Eq.3)

where 𝑎(𝑡) = [𝑋^2^(𝑡)+𝑌^2^(𝑡)]^1/2^ is the envelope of the signal and 𝜃(𝑡) = tan^−1^(𝑌/𝑋) represents its instantaneous phase. The instantaneous frequency of the Hilbert spectrum can now be defined using:

$w=\frac{d\theta}{\mathrm{dt}}$ (Eq.4)

By applying the Hilbert transform to individual IMFs, the EMD decomposed the original signal $x\left( t \right)$ (omitting the residue) can be expressed as

$x\left( t \right)=\sum_{i=1}^{K} a_{i}(t)exp\left\{ i\int w_{j}(t)dt \right\}$ (Eq.5)

### Calculation of HWF and HWMF

Next, considering the total number of 𝑁 time points of the input signal *x*(𝑡), the Hilbert weighted frequency ${HWF}_{j}$ of each IMF is obtained by taking the weighted mean of instantaneous frequencies $w_{j}\left( i \right)$ using:

${HWF}_{j}=\frac{\sum_{i=1}^{N} a_{j}^{2}\left( i \right)w_{j}(i)}{\sum_{i=1}^{N} a_{j}^{2}\left( i \right)}$ (Eq.6)

And the Hilbert weighted mean frequency of the original signal (𝑡) can be obtained by the weighted contribution of the mean frequencies of the individual IMFs {${HWF}_{j}$},=1 to 𝐾, by

$HWMF=\frac{\sum_{j=1}^{N} \left\| a_{j} \right\|{HWF}_{j}}{\sum_{j=1}^{N} \left\| a_{j} \right\|}$ (Eq.7)
