# Supplementary figures and images for: Frequency-Dependent Modulation of Regional Synchrony in the Human Brain by Eyes Open and Eyes Closed Resting-States
Source: PLoS One. 2015 Nov 6;10(11):e0141507. doi: 10.1371/journal.pone.0141507 (PMC4636261; doi:10.1371/journal.pone.0141507)

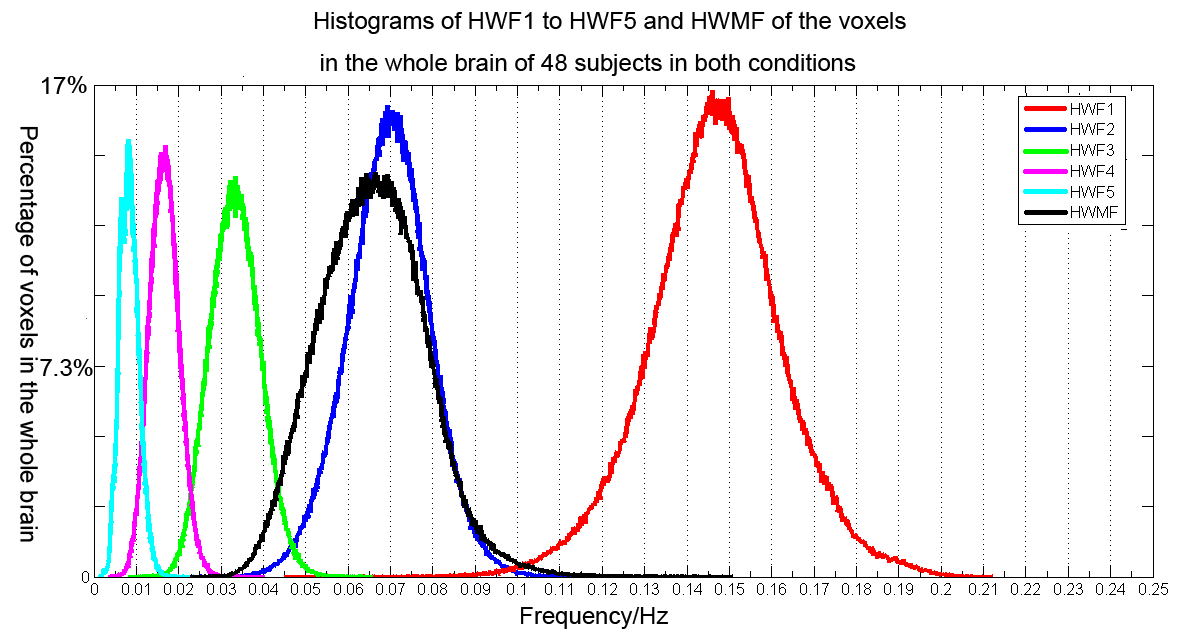

Supplement: S1 Fig — The histograms of HWF of IMF1 to IMF5 (color-coded by red, blue, green, pink, and cyan respectively) were determined from all the voxels in the whole brains across all the 48 subjects and across both the EO and EC conditions. Heights of the histograms represent the percentage of voxels in the whole brain whose HWF equals that frequency on the horizontal axis. (TIF) [file pone.0141507.s002.tif]

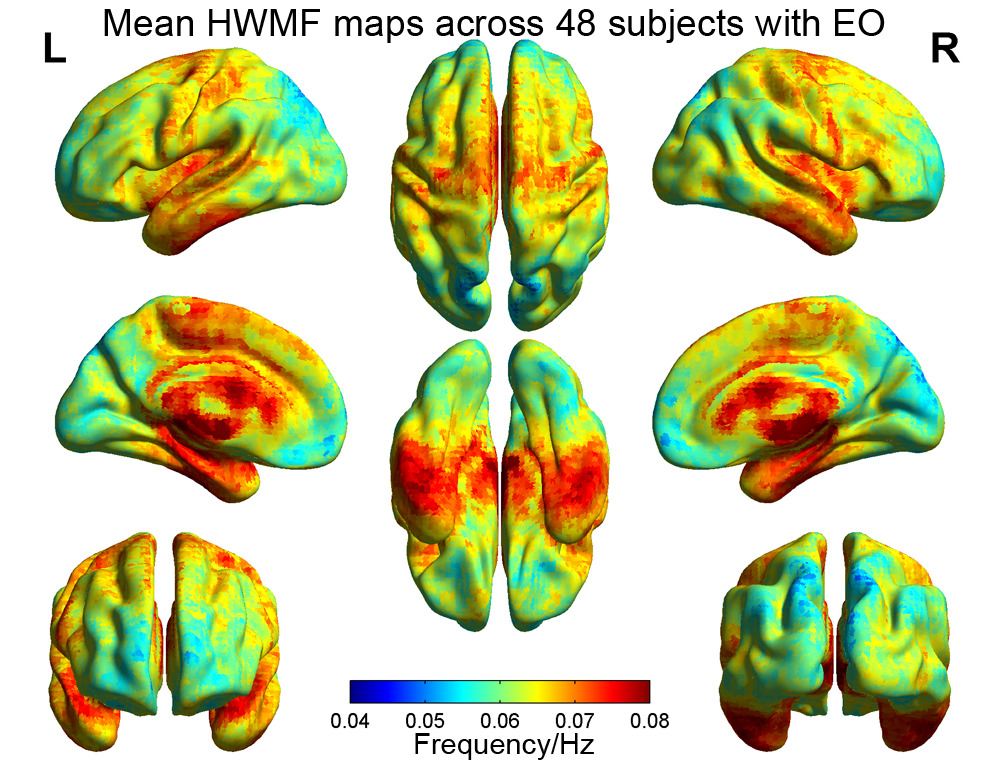

Supplement: S2 Fig — Voxels whose HWMF are equal or less than 0.04 Hz are color-coded as dark blue, voxels whose HWMF are equal or higher than 0.80 Hz are color-coded as dark red. (TIF) [file pone.0141507.s003.tif]

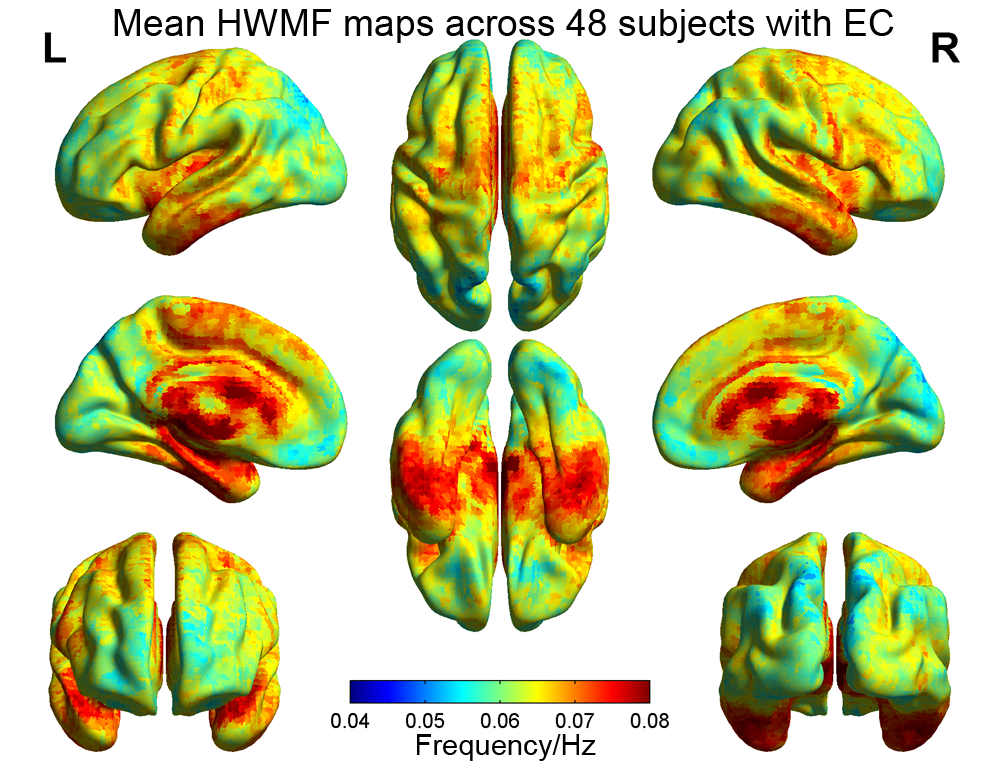

Supplement: S3 Fig — Voxels whose HWMF are equal or less than 0.04 Hz are color-coded as dark blue, voxels whose HWMF are equal or higher than 0.80 Hz are color-coded as dark red. (TIF) [file pone.0141507.s004.tif]

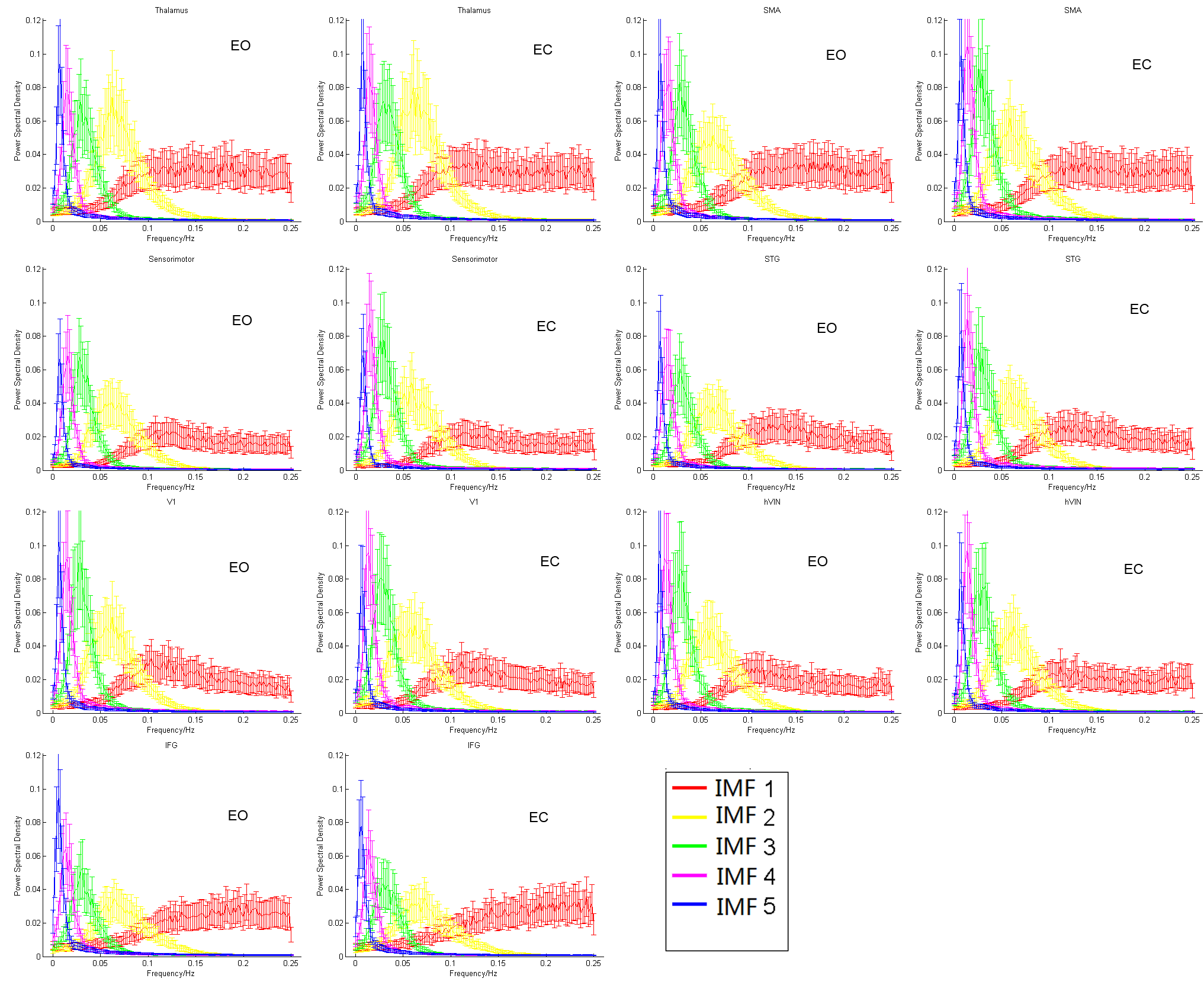

Supplement: S4 Fig — The IMF time courses of all the voxels within each of the ROIs were extracted and averaged to get a mean IMF time course from which the power spectrum of that ROI of that subject was calculated. For each IMF of each ROI, the power spectrums were then averaged across the 48 subjects. (TIF) [file pone.0141507.s005.tif]
