# Supplementary material for: Frequency-Dependent Modulation of Regional Synchrony in the Human Brain by Eyes Open and Eyes Closed Resting-States
Source: PLoS One. 2015 Nov 6;10(11):e0141507. doi: 10.1371/journal.pone.0141507 (PMC4636261; doi:10.1371/journal.pone.0141507)
Supplement: S1 Table — Alphasim corrected, voxel-level p < 0.01, cluster size > 68 voxels, overall false positive P < 0.01. Brain areas with significant changes of HWMF and ReHo are labeled with orange and green backgrounds respectively. (DOCX) [file pone.0141507.s006.docx]

# Supplementary materials

## Tables

| Brain region name | side | BA | ReHo | | | | Frequency | | |
| --- | --- | --- | --- | --- | --- | --- | --- | --- | --- |
|  |  |  | cluster size | peak voxel MNI | | T statistic | cluster size | peak voxel MNI | T statistic |
| Thalamus | R | - | 76 | | 12, -15,15 | -4.5498 | 81 | 15,-15,12 | 4.5034 |
| Thalamus | L | - | 70 | | -15,-18,15 | -4.2195 | 69 | -8,-17,8 | 4.5358 |
| Supplementary motor area | L,R | 6 | 112 | | 12,-12,69 | -4.7943 | 105 | 0,-24,54 | 4.5303 |
| Sensorimotor cortex | L | 6,4 | 310 | | -63,-9,24 | -4.7869 | 302 | -51,-15,39 | 4.5277 |
| Sensorimotor cortex | R | 6,4 | 286 | | 60,-12,39 | -4.4264 | 288 | 48,-9,36 | 4.4865 |
| Superior Temporal Gyrus | L | 6 | 267 | | -49,-19,12 | -4.5274 | 265 | -62,-15,4 | 4.5073 |
| Superior Temporal Gyrus | R | 48 | 280 | | 48,-30,6 | -4.4133 | 274 | 48,-33,6 | 4.4994 |
| Calcarine | L,R | 17 |  | |  |  | 132 | -3,-72,12 | 4.1541 |
| Middle Occipital gyrus | L | 19 |  | |  |  | 205 | -24,-78,27 | 4.6635 |
| Middle Occipital gyrus | R | 19 |  | |  |  | 224 | 39,-69,24 | 4.5728 |
| Inferior forntal cortex | L | 10,11 |  | |  |  | 286 | -9,63,-15 | 4.1684 |
| Inferior forntal cortex | R | 10,11 |  | |  |  | 267 | 15,51,0 | 4.5672 |

**S1 Table.** Brain areas with significant (Alphasim corrected, voxel-level *p* < 0.01, cluster size > 68 voxels, overall false positive *P* < 0.01) changes of Frequency (orange background) or ReHo (green background) between EO and EC conditions.
